# Population structure and history of the Welsh sheep breeds determined by whole genome genotyping

**DOI:** 10.1186/s12863-015-0216-x

**Published:** 2015-06-20

**Authors:** Sarah E. Beynon, Gancho T. Slavov, Marta Farré, Bolormaa Sunduimijid, Kate Waddams, Brian Davies, William Haresign, James Kijas, Iona M. MacLeod, C. Jamie Newbold, Lynfa Davies, Denis M. Larkin

**Affiliations:** Institute of Biological, Environmental and Rural Sciences, Aberystwyth University, Penglais, Aberystwyth, Ceredigion SY23 3DA UK; Royal Veterinary College, University of London, Royal College Street, London, NW1 0TU UK; Victorian Department of Environment and Primary Industries, Bundoora, VIC 3083 Australia; Commonwealth Scientific and Industrial Research Organisation (CSIRO), 306 Carmody Road, St Lucia, QLD 4067 Australia; Faculty of Veterinary and Agricultural Sciences, University of Melbourne, Melbourne, VIC 3010 Australia; Hybu Cig Cymru, Meat Promotion Wales, Tŷ Rheidol, Parc Merlin, Aberystwyth, SY23 3FF UK

**Keywords:** Ovis aries, Welsh native breeds, Selection, Population structure, Genotyping, Demography, Selective breeding, SNP, Sequencing, Linkage disequilibrium

## Abstract

**Background:**

One of the most economically important areas within the Welsh agricultural sector is sheep farming, contributing around £230 million to the UK economy annually. Phenotypic selection over several centuries has generated a number of native sheep breeds, which are presumably adapted to the diverse and challenging landscape of Wales. Little is known about the history, genetic diversity and relationships of these breeds with other European breeds. We genotyped 353 individuals from 18 native Welsh sheep breeds using the Illumina OvineSNP50 array and characterised the genetic structure of these breeds. Our genotyping data were then combined with, and compared to, those from a set of 74 worldwide breeds, previously collected during the International Sheep Genome Consortium HapMap project.

**Results:**

Model based clustering of the Welsh and European breeds indicated shared ancestry. This finding was supported by multidimensional scaling analysis (MDS), which revealed separation of the European, African and Asian breeds. As expected, the commercial Texel and Merino breeds appeared to have extensive co-ancestry with most European breeds. Consistently high levels of haplotype sharing were observed between native Welsh and other European breeds. The Welsh breeds did not, however, form a genetically homogeneous group, with pairwise *F*_ST_ between breeds averaging 0.107 and ranging between 0.020 and 0.201. Four subpopulations were identified within the 18 native breeds, with high homogeneity observed amongst the majority of mountain breeds. Recent effective population sizes estimated from linkage disequilibrium ranged from 88 to 825.

**Conclusions:**

Welsh breeds are highly diverse with low to moderate effective population sizes and form at least four distinct genetic groups. Our data suggest common ancestry between the native Welsh and European breeds. These findings provide the basis for future genome-wide association studies and a first step towards developing genomics assisted breeding strategies in the UK.

**Electronic supplementary material:**

The online version of this article (doi:10.1186/s12863-015-0216-x) contains supplementary material, which is available to authorized users.

## Background

Domestication and subsequent artificial selection for economically or aesthetically important traits and breed formation have substantially altered the genetics and diversity of animal populations [1, 2]. A central goal within contemporary genetics of livestock species is the detection of causative mutations affecting traits with economic value. Whilst this analysis is complex, particularly where traits are controlled by multiple loci (quantitative trait loci; QTLs), the results can be invaluable in developing effective breeding programmes. With the advent of new sequencing and genotyping technologies, the large-scale detection of QTLs in some livestock populations became feasible [3]. Such analysis is aided by a detailed knowledge of the demographic history and genetic structure of these populations [4, 5]. Studies of demographic history within and between breeds also enable the estimation of the number of genetic markers required to effectively predict individual breeding values (i.e., genomic selection) and indicate how transferable such predictions would be to other populations [6, 7].

Understanding the structure and origin of livestock populations (breeds) is crucial for the success of genomic selection, targeted marker-assisted breeding, and QTL detection through genome-wide association studies (GWAS). In particular, studies focused on rare breeds give us a chance to identify variation and understand the biological mechanisms that enable these breeds to survive in different local environments. The development of intensive farming systems based on a small number of commercial breeds has gradually led to a decrease in diversity within some livestock populations [8, 9]. Cataloguing the variation within the rarer locally adapted breeds is therefore critical in ensuring this important genetic resource is not lost [10].

Unlike cattle, sheep have retained a relatively high level of genetic diversity [11]. A recent study of 2819 individuals from 74 diverse sheep breeds collected from Asia, Africa, the Caribbean, North and South America, Europe, Australia and New Zealand demonstrated that domestic sheep breeds have higher effective population sizes (*N*_*e*_ ranging from 100 to 1317) than the majority of contemporary cattle breeds (*N*_*e*_ = 99 for Holstein and *N*_*e*_ = 97 for Hereford) [12]. A high degree of haplotype sharing among sheep breeds also suggested a common origin of breeds [11]. This contrasts with data from cattle (*Bos indicus* and *Bos taurus*) and pig (*Sus scrofa*), which provide clear evidence of two domestication events [13, 14]. A high level of admixture was demonstrated within the European sheep, with Merino breeds having high co-ancestry with most other breeds. These findings likely reflect the widespread use of Merino sires throughout Europe after the Middle Ages [11]. While the comprehensive study of 74 breeds distributed worldwide provided important insights into the domestication history and formation of sheep breeds globally, more focused studies involving larger numbers of local breeds are required to ensure the efficacy of future breeding programmes. With this in mind, we performed a detailed study of sheep diversity and breed structure in Wales.

It is believed that sheep were first introduced into the British Isles by Neolithic settlers [15]. After the Roman conquest, additional breeds with superior wool quality were brought into the country from Southern Europe [16]. Sheep were primarily seen as wool animals and this continued to be the case until the Middle Ages, when farmers began to breed sheep for milk and meat instead [15]. The industrial revolution led to population growth and an increased demand for food, including meat [15]. To meet this rise in demand, the sheep farming community turned its attention to selecting and breeding animals for carcase and meat quality, a trend which has continued to the present day [15]. The breeds found in the UK (including Wales) today are therefore the result of breeding to produce good quality meat animals. Unlike lowland meat breeds, such as the Texel, Welsh breeds have also been selected for hardiness to enable them to survive in the harsh Welsh landscape.

In this study, we analysed a data set composed of native Welsh sheep in the context of a set of worldwide breeds. We used the Illumina Ovine single nucleotide polymorphism (SNP) array containing 54,241 SNPs to genotype individuals from 18 breeds native to Wales and combined our data with that from an additional 74 breeds provided by the International Sheep Genome Consortium [11, 17]. This data set comprised 2819 individuals, mainly from breeds of European origin, but also included 15 Asian, 6 African and 6 American breeds [11]. In addition, individuals of the same breed from different geographical locations were also included in the HapMap data set [12], with Texel samples from Scotland, New Zealand and Germany and Merino samples from China and Australia. The breeds sampled included wool, milk and meat sheep. We aimed to build on this established resource and use it as a reference for interpreting population structure and genetic diversity within the native Welsh breeds, as well as for developing hypotheses about their relationships with breeds worldwide.

To enable comparisons with similar studies of this type, we used standard analytical techniques. Several complementary model-based and assumption-free clustering methods (i.e., multidimensional scaling, STRUCTURE and phylogenetic analysis) were used to avoid artefacts and assess the robustness of the detected patterns. In addition, we estimated the extent of haplotype sharing at different scales in order to distinguish between older and more recent breed relationships. Finally, we estimated effective population sizes from linkage disequilibrium (LD) and used whole genome sequence data to reconstruct historical demographic trends. The resulting patterns are informative about the effects of trade and migration on the development of extant Welsh breeds.

## Methods

### Sample collection

We located flocks of 18 native Welsh sheep breeds using breed society and flockbook information (Table 1). Where pedigree details were available, we attempted to avoid sampling of individuals known to be closely related (e.g. siblings, parent and offspring). Animals of different ages and family groups were selected as identified by the farmer. The presence of some close relatives in the sample set cannot be excluded, however. Sample size ranged from 6 to 24 and was dependant on availability of suitable pedigree flocks. Breeds with lower sample numbers (<10) were retained but the estimated values of effective population size and linkage disequilibrium decay should be treated with caution (Table 1). Collection of blood (maximum volume = 10 ml) was carried out by superficial venepuncture using sterile 10-ml BDK2EDTA Vacutainers® (BD, Becton, Dickinson and company, Oxford, UK). Buffy coat preparations were generated from samples on the day of sampling through centrifugation for 30 min at 450 g and 4 °C. For each sample, buffy coat or whole blood was stored at −80 °C until further use.

**Table 1:** Single nucleotide polymorphism, diversity, inbreeding and linkage disequilibrium within Welsh breeds

Breed	*No.* ^a^	*F* ^b^	*P*_*n*_^c^	*H*_*e*_^d^	*N*_*e*_^e^	No. sampling locations
Tregaron Welsh Mountain	6	0.024	0.911	0.361	117	1
Improved Welsh Mountain	15	0.035	0.968	0.377	649	1
Llandovery White Faced	24	0.046	0.970	0.377	806	1
Dolgellau Welsh Mountain	8	0.052	0.920	0.358	158	1
Hill Flock Welsh Mountain	24	0.055	0.965	0.367	431	1
Talybont Welsh Mountain	24	0.060	0.946	0.354	188	1
Welsh Hardy Speckled Faced	24	0.062	0.969	0.374	603	2
Brecknock Hill Cheviot	24	0.064	0.930	0.350	737	1
Badger Faced	24	0.066	0.973	0.380	825	3
Lleyn	22	0.077	0.934	0.351	207	2
Hill Radnor	21	0.118	0.942	0.350	198	2
South Wales Welsh Mountain	18	0.119	0.820	0.341	141	1
Beulah	23	0.123	0.900	0.324	102	1
Llanwenog	22	0.139	0.913	0.337	149	2
Clun Forest	17	0.143	0.884	0.328	104	1
Balwen	15	0.160	0.920	0.323	94	1
Black Welsh Mountain	24	0.206	0.900	0.327	89	2
Kerry Hill	18	0.213	0.869	0.306	88	1
Total/Average	353	0.098	0.924	0.349	316	1.4

^a^No. of individuals genotyped from each breed

^b^Inbreeding coefficient

^c^Proportion of polymorphic loci within a breed

^d^Expected heterozygosity

^e^Effective population size estimated from linkage disequilibrium

### DNA extraction and SNP genotyping

DNA extraction was performed using the Qiagen Blood and Cell Culture Midi Kit (Qiagen Ltd, Manchester, UK), following the blood sample preparation and extraction protocol outlined in the Qiagen Genomic DNA Handbook [18]. DNA quality and quantity were determined using a NanoDrop 2000c (Thermo Scientific, Wilmington, DE, USA). High quality samples (i.e., having concentrations of at least 50 ng/μl and A260/280 ratios of ca. 1.8) were then subjected to array genotyping using the Illumina OvineSNP50 manual protocol (Illumina Inc., San Diego, CA). Genotypes were called using the GenomeStudio software (Illumina), and samples with call rates of less than 95 % were excluded from further analyses. A pedigree (.ped) file containing the genotype calls, sample and family identifiers and a map (.map) file containing the chromosomal location and identifier for each SNP were generated using GenomeStudio and imported into the PLINK whole genome analysis toolkit [19] for further processing. In PLINK, SNPs with minor allele frequency (MAF) < 0.01 were removed from the data set, along with a subset of those previously identified as showing atypical chromosome X clustering or inconsistency between sequencing techniques (Table 2) [11].

**Table 2:** SNP genotyping and filtering statistics

Statistics	Welsh data set	Combined data set
SNPs genotyped	51,135	51,135
MAF^a^	50,741	51,036
HapMap quality filter^b^	48,640	48,935
Autosomal SNPs^c^	46,266	46,561
LD pruned^d^	11,527	25,254

^a^Minor allele frequency >0.01

^b^Removal of SNPs identified as being of poor quality as defined by the International Sheep Genome Consortium HapMap project [11]

^c^Removal of SNPs on the sex chromosomes

^d^Removal of one SNPs from each pair where *r* ^*2*^ > 0.05 within 50 SNP blocks

### Single nucleotide polymorphism, diversity and linkage disequilibrium within Welsh breeds

Inbreeding coefficients (*F*) for each individual and the proportion of polymorphic loci (*P*_*n*_) in each breed were calculated using the PLINK *--hardy*, *--freq* and *--het* commands*.* Estimates of expected heterozygosity (*H*_*e*_) at each locus were calculated using the *--hardy* command in PLINK and the mean was calculated for each breed. To calculate pairwise differentiation (*F*_ST_) between different breeds we used Eigensoft (v 5.0.1) [20, 21]. We estimated linkage disequilibrium (LD) by calculating *r*^*2*^ for all pairs of SNPs with MAF ≥ 0.10 that were located within 1 Mb of each other using the *--maf 0.1 --ld-window-r2 0 --ld-window-kb 1000* options in PLINK. Based on these *r*^*2*^ values, we then estimated the recent effective population size (*N*_*e*_) for each breed and across all Welsh breeds using the method described by Tenesa et al. [22] and following the assumptions made by Kijas et al. [11]. Briefly, we assumed that 1 Mb = 1 cM across the sheep genome and fitted the non-linear regression model of Tenesa et al. [22] to *r*^*2*^ values corrected for sample size to obtain *N*_*e*_ estimates. In addition, we calculated a relative measure of haplotype sharing (*r*) among breeds following the methodology described by Kijas et al. [11]. This was done for intervals of 0–10Kb, 10–25Kb, 25–50Kb and 50–100Kb for all 48,922 SNPs that met the initial filtering criteria to make our analysis comparable with the results of the sheep HapMap project [12].

### Population structure and phylogenetic analyses

Population structure was characterised using: 1) model-based clustering, 2) assumption-free multidimensional scaling (MDS) and 3) distance-based phylogenetic analysis. To ensure that analyses would not be distorted by the presence of SNPs in strong LD, the *--indep* command in PLINK was used to prune the SNPs that passed the initial filtering steps. This was achieved by removing one locus from each pair for which LD (*r*^*2*^) exceeded 0.05 within 50-SNP blocks. We used PGDSpider (v2.0.4.0) [23] to convert these data into a format suitable for input into the clustering and stratification program STRUCTURE (v2.3.4) [24–27]. Initial runs of STRUCTURE were carried out assuming between 1 and 18 groups (K), with a burnin period of 5000 cycles and 10,000 data collection Markov chain Monte Carlo (MCMC) cycles. Five runs were performed for each value of K. A subset of longer runs was also performed, each run having a burnin period of 20,000 cycles and 50,000 MCMC data collection cycles, to confirm the patterns detected through shorter runs. Separate runs were aligned using the CLUMPP program (v 1.1.2) [28]. To visualise the subpopulation membership coefficients for each individual we used DISTRUCT [29]. As an assumption-free illustration of the differentiation between breeds, multidimensional scaling (MDS) analysis was performed using the *--cluster* and *--meds-plot* commands within the PLINK toolkit. Results from the MDS analysis were visualised in R [30]. Neighbour joining trees were generated from an identity by state distance matrix using the NEIGHBOR program in PHYLIP [31]. The resultant trees were visualised using FigTree [32].

To identify relationships between Welsh native sheep and other breeds worldwide, we combined our data set with that generated by the International Sheep Genome Consortium HapMap project. This data set comprised 2819 individuals from 74 breeds distributed worldwide and genotypes for a set of SNPs that were consistent with our data [11]. These data sets were combined using the PLINK toolkit *--merge* command. The combined data were then filtered using the same quality and LD pruning criteria as for the Welsh breed data set, resulting in a subset of 25,254 SNP that we used for downstream analyses as described above for the 18 Welsh breeds (Table 2). This was comparable to the International Sheep Genome Consortium HapMap study, which utilised 22,678 SNPs obtained after similar filtering but for a smaller set of animals [11].

### Historical demographic trends from whole genome sequence data

To further understand the demographic history of the Welsh mountain breeds we used two methods of demographic inference. We refer to the methods used as the pairwise sequentially Markovian coalescent model (PSMC), developed by Li and Durbin [33] and the Haplotype homozygosity (HHn) method developed by MacLeod et al. [34, 35]. Both methods utilised Illumina (~12 × coverage) whole genome sequence from single individuals of the Hardy Speckled Faced, Dolgellau and Tregaron Welsh Mountain breeds (SRA accession numbers: SRX150321, SRX150316 and SRX150322, respectively) [36]. Additional details on sequencing methodology are described by Heaton et al. [36]. The Burrows-Wheeler Aligner (BWA)-backtrack algorithm was used to map reads against the sheep reference assembly v3.0 (available at http://www.livestockgenomics.csiro.au/sheep/) using default parameters [37]. Around 85 % of reads were aligned and unmapped reads or reads mapping to multiple positions in the reference were removed.

The PSMC method applies a hidden Markov model to interrogate the genome-wide pattern of heterozygosity, whereas the HHn method relies on a summary statistic that describes the distribution of genome-wide runs of homozygosity (RoH). These two demographic inference methods were chosen because they exploit whole genome sequences, which allows for better resolution of demography going back in time, than using a restricted number of autosomal loci or mitochondrial DNA. Although the PSMC model has been used widely across a range of diploid species, the resolution of population size (*N*_*e*_) inference in more recent time is generally limited [33]. There is some evidence that the HHn method may have better sensitivity than PSMC to infer recent time demography [34]. Therefore it was of interest to compare the inferred demography from both methods.

Although the mathematical approaches differ, both demographic inference methods require accurate sequence calls of only the heterozygous SNPs in the sequence of individuals. It is critical for both methods to have good estimates of the rates of two types of errors in heterozygous SNP calls. The false negative error rate is the proportion of real heterozygous sites that are missed, whereas the false positive rate is the proportion of all base pairs that are erroneously called heterozygous.

Initial SNP calling from the whole genome sequence of the three individuals was carried out using the software GigaBayes [3, 34, 38]. Heterozygous SNPs were included in the analysis only if (i) the minimum read depth exceeded four reads for each allele, (ii) the ratio of minor allele reads to total allele reads was at least 0.25 and (iii) the total read depth was less than twice the average read depth across the genome. The false negative and false positive error rates were estimated following MacLeod et al. [34] by comparisons with independent Illumina OvineSNP50 chip genotypes from the set described above. The false negative rate was corrected for by scaling the mutation rates for the demographic inference [33, 34]. Briefly, false negatives are assumed to occur randomly across the genome and therefore their effect on the patterns of heterozygosity/homozygosity is equivalent to lowering the true mutation rate (*μ*). The false negative rate (expressed as the proportion of heterozygous positions missed) was estimated from the concordance of heterozygous SNP chip genotypes with the same SNP positions in the sequence data. The scaled mutation rate is then: *μ*_*scaled*_ = *μ*(1 − *q*), where *μ* is the mutation rate per base pair per generation and *q* is the estimated false negative error rate.

To correct for potential bias from false positive errors, we applied the MacLeod et al. [34] method using an error correction window length of 10 × [1/false positive error rate] base pairs. This correction method randomly removed ten heterozygous positions from each non-overlapping window across the genome, where the window size matched the expected segment size estimated to contain ten false positive errors (i.e. specific to estimated error rates in each genome). This was carried out prior to estimating the observed distribution of RoH. This has been previously demonstrated to help restore the distribution of RoH closer to the error-free distribution and therefore remove or reduce bias in the more recent time *N*_*e*_ inference [33]. In the absence of direct estimates of the mutation rate in sheep, constant genome-wide mutation and recombination rates of 1 × 10^−8^ were assumed, as for cattle demographic estimates [34]. Several recent estimates of mutation rates in humans are close to 1 × 10^−8^ [39–41]. As estimates of *N*_*e*_ obtained through both the HHn and PSMC methods depend very strongly on the assumed rates of mutation and sequencing errors, we used these methods only to detect historical demographic trends. Our sensitivity analyses demonstrated that, unlike estimates of *N*_*e*_, demographic trends were not affected by our modelling assumptions (see Results). As demonstrated in Additional file 1: Figure S1 and Additional file 2: Figure S2, if mutation rate is assumed to be 2 × 10^−8^ rather than 1 × 10^−8^, the overall pattern of demography remains the same, but *N*_*e*_ is reduced by about half and the timing of all changes in *N*_*e*_ shifts to slightly more recent periods. If the false negative error rates are incorrectly estimated this will also affect the demography in a similar way because the actual mutation rate used for inference is scaled by (1-false negative error rate).

A range of additional parameter settings were tested for PSMC inference and those found to be optimal for these sheep sequences were: number of iterations = 25, T_max_ = 12, rho = 2.5, and estimates of the stepwise population size parameters were allocated to atomic time intervals of 4*1 + 15*2 + 1*6. For both methods, an average sheep generation interval of 4 years and a total autosomal genome length of 2,452,040,444 base pairs were assumed. The PSMC bootstrapping method (100 samples) was used to calculate confidence intervals, given the parameters described above. For the HHn method, mutation and recombination rates were fixed at 1 × 10^−8^. Time intervals were not pre-set because the period of generations for stepwise *N*_*e*_ may change with each demography tested if this results in a better fit of predicted and observed distributions of RoH. A large number of demographic models were tested and the “best fit” model was determined as one which met the threshold goodness of fit criteria (δ ≤ 0.001) between the predicted and observed HHn statistics (RoH of 1 bp to 1 Mb). The upper and lower bounds for *N*_*e*_ (i.e. where δ ≤ 0.001) were then sequentially calculated for each stepwise period of constant *N*_*e*_, while *N*_*e*_ in all other time intervals was fixed as for the “best fit” model. Therefore, these upper and lower limits indicate the possible range of *N*_*e*_ for a single time period, while all other time periods remain as per the best fit model [34].

## Results

### Polymorphism, diversity and linkage disequilibrium within Welsh breeds

The set of 50K Illumina SNPs was found to be highly informative in all Welsh breeds (Table 1). The proportion of polymorphic loci (*P*_*n*_) within a breed ranged from 0.820 (South Wales Welsh Mountain) to 0.973 (Badger Faced), with a mean 0.924. Similarly, expected heterozygosity (*H*_*e*_) was relatively high in all Welsh breeds (mean = 0.349, range = 0.306–0.380) [42]. As expected, weak to moderate rates of inbreeding (*F*) were detected in all breeds, with a mean 0.098 and range from 0.024 (Tregaron Welsh Mountain) to 0.213 (Kerry Hill). Consistent with the relatively high within-breed diversity and relatively low levels of inbreeding, LD for common SNPs (MAF ≥ 0.10) decayed relatively rapidly, with average *r*^*2*^ dropping to less than 0.2 within 30–80Kb in most breeds and within 20Kb across the combined data for 18 Welsh breeds (Additional file 3: Figure S3). Consequently, LD-based estimates of recent *N*_*e*_ calculated using the Illumina OvineSNP50 chip data were relatively high for a domesticated animal (mean = 316, range = 88–825) (Table 1).

### Ancestry of the native Welsh breeds

**Fig. 1:**
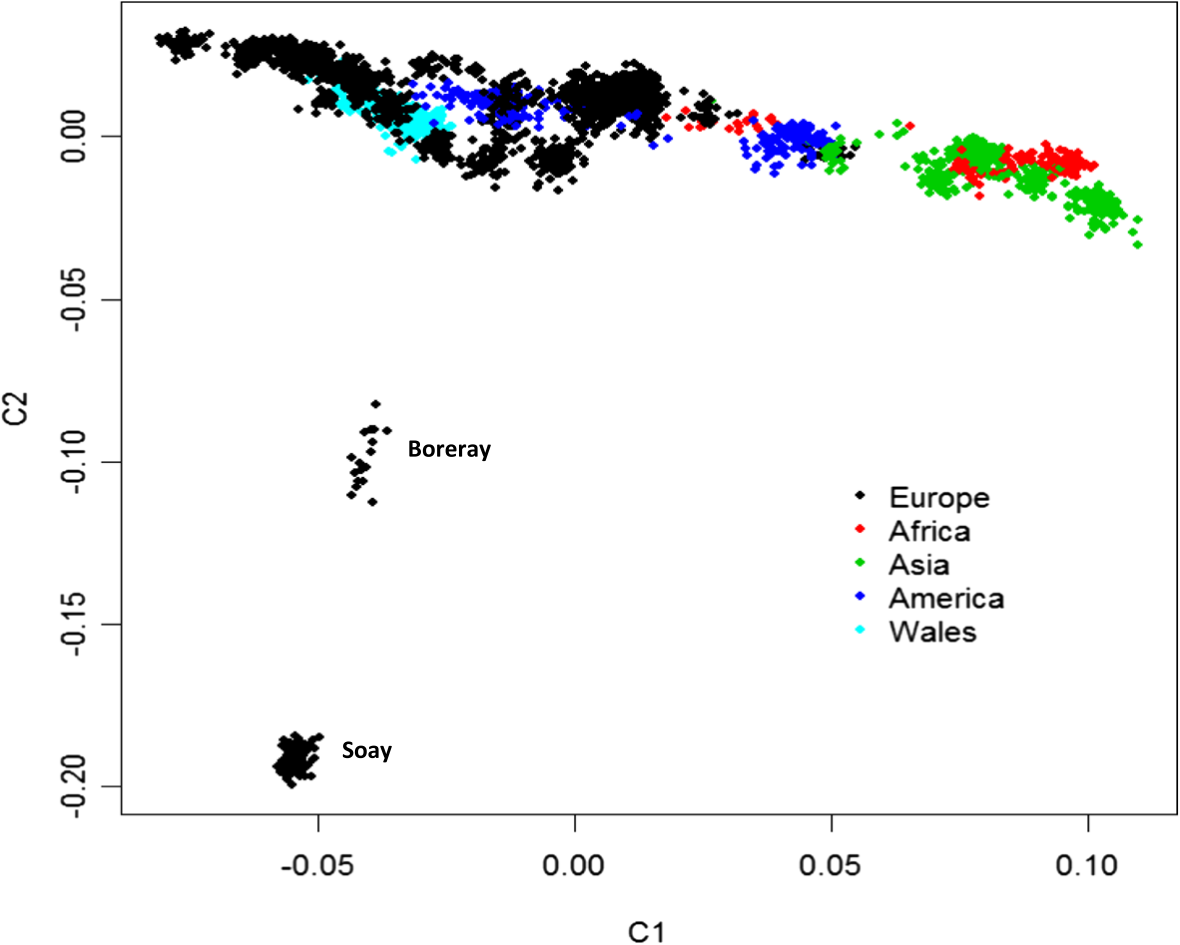
Clustering of Welsh and worldwide sheep breeds based on multi-dimensional scaling of genotype data. Individuals are shown in the context of the International Sheep Genome Consortium HapMap data set of 74 breeds [11]

To identify ancestral relationships between the Welsh and other worldwide breeds, we analysed our data set jointly with that of the International Sheep Genome Consortium HapMap project [11]. As expected, the first two principle components identified through MDS separated African and Asian breeds from a central cluster of breeds with European origins, including all Welsh breeds (Fig. 1). Also consistent with previous findings, the only European breeds that formed separate clusters at this level of resolution were Soay and Boreray. Similar patterns were detected through the STRUCTURE analyses (Additional file 4: Figure S4), which also showed clear separation of European, African and Asian breeds.

**Fig. 2:**
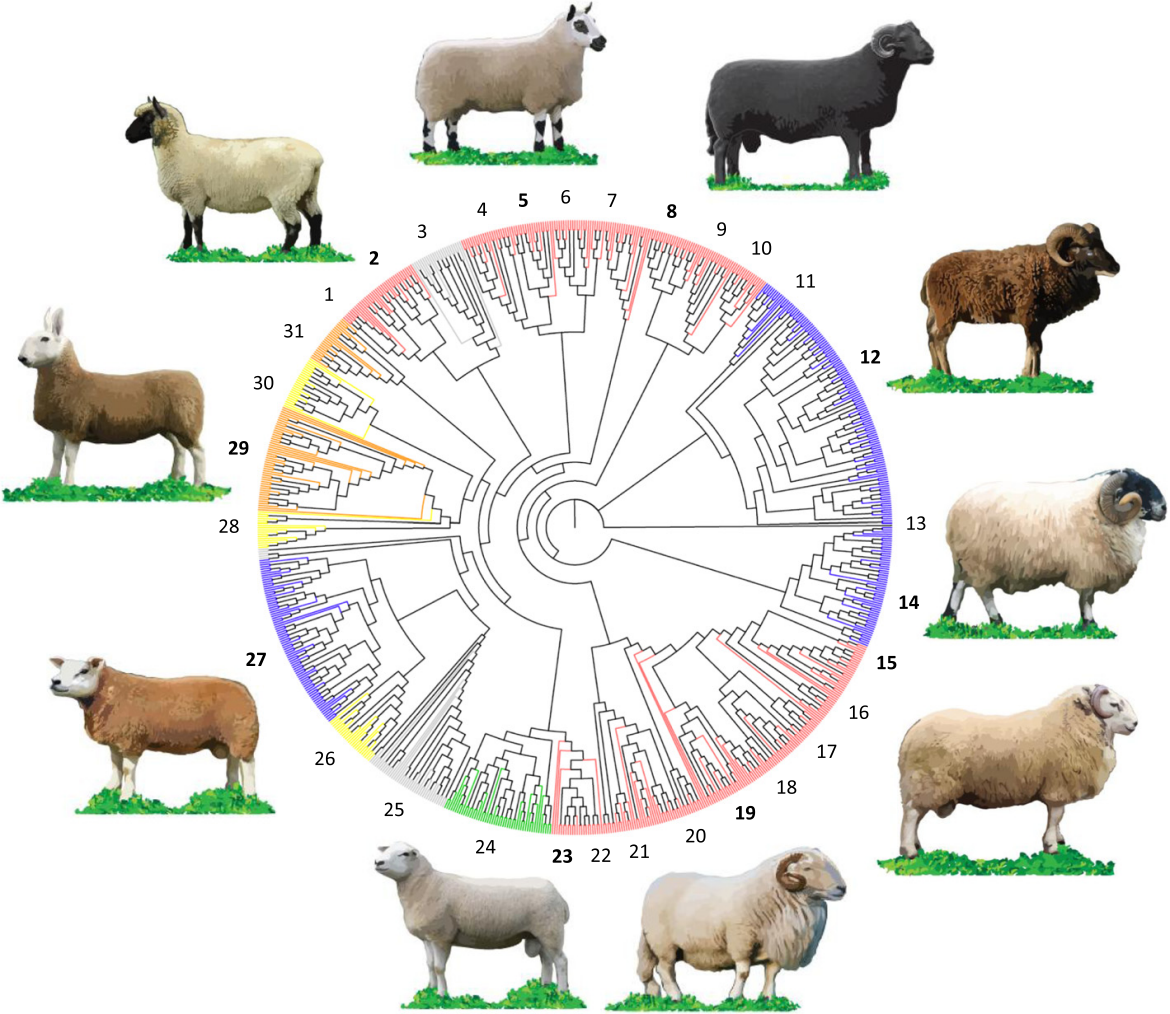
Phylogeny of 18 native Welsh sheep breeds and their most closely related European breeds. Welsh breeds are shown in red, European in grey, Australian/New Zealand in yellow, Scottish in blue, English in yellow and Irish in green. An Indian Garole individual was used to provide an outgroup (no. 13). 1) Llanwenog, 2) Clun Forrest, 3) Black Headed Mutton, 4) Beulah, 5) Kerry Hill, 6) Welsh Hardy Speckled Faced, 7) Hill Radnor, 8) Black Welsh Mountain, 9) Balwen, 10) Badger Faced, 11) Boreray, 12) Soay, 13) Indian Garole - Outgroup, 14) Scottish Blackface, 15) Brecknock Hill Cheviot, 16) Llandovery White Faced, 17) Talybont Welsh Mountain, 18) Improved Welsh Mountain, 19) South Wales Welsh Mountain, 20) Hill Flock Welsh Mountain, 21) Dolgellau Welsh Mountain, 22) Tregaron Welsh Mountain, 23) Lleyn, 24) Galway, 25) German Texel, 26) New Zealand Texel, 27) Scottish Texel, 28) Australian Coopworth, 29) Border Leicester, 30) New Zealand Romney, 31) Wiltshire. Breed names shown in bold correspond to the adjacent breed images

**Fig. 3:**
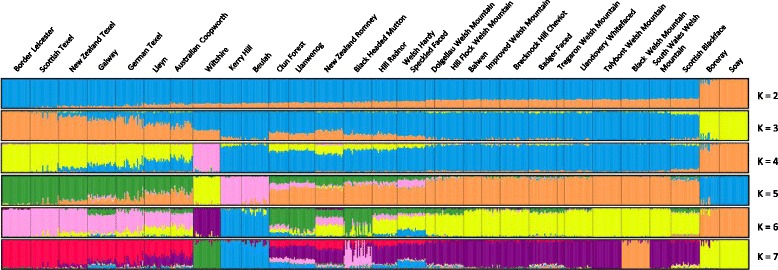
Model-based clustering of 18 native Welsh sheep breeds and their most closely related European breeds. Analysis was performed using the program STRUCTURE, with K representing the assumed number of populations

Based on our initial phylogenetic analysis (Additional file 5: Figure S5), a subset of the International Sheep Genome Consortium breeds found to be most closely related to the Welsh breeds were selected for subsequent, higher-resolution phylogenetic analyses (Fig. 2). This group consisted of the 18 Welsh breeds and 12 additional breeds of European ancestry, including Texel individuals from different locations, the Border Leicester, Galway, Wiltshire, and New Zealand Romney breeds. An Indian Garole individual was included as an outgroup to root the tree. Neighbour-joining clustering of these 31 breeds identified a major cluster of the Welsh Mountain breeds (with the exception of the Black Welsh Mountain breed). In addition, the Welsh Lleyn breed clustered with the Galway breed from Ireland, whereas the European Black Headed Mutton breed clustered with the Welsh Llanwenog and Clun Forest breeds (Fig. 2). Model based clustering of the same set of 31 breeds resulted in the detection of similar patterns (Fig. 3). In addition, these STRUCTURE analyses indicated the distinctness of the Soay and Boreray breeds (i.e., at *K* = 2), the Wiltshire breed (i.e., at *K* = 4), as well as the group consisting of the Welsh Beulah and Kerry Hill breeds (i.e., at *K* = 5). The first of the Welsh breeds observed to form a potential individual subpopulation was the Black Welsh Mountain breed.

On analysing haplotype sharing over intervals of increasing length (0–10Kb, 10–25Kb, 25–50Kb and 50–100Kb), we identified the 10 International Sheep Genome Consortium HapMap breeds showing the highest levels of sharing with the Welsh breeds. The Texel (German and Scottish), Merino (Australian, Australian Poll, and Australian Industry), Australian Suffolk, Gulf Coast Native, Lacaune (Milk and Meat) and Scottish Blackface breeds had consistently high levels of haplotype sharing with the Welsh breeds over at least three of the four intervals studied (Additional file 6: Figure S6). In addition, the Galway, Finnsheep and Rasa Aragonesa breeds showed high levels of haplotype sharing at two of the four intervals. Interestingly, the highest-resolution haplotype sharing analysis (0–10Kb), which presumably reflects the oldest historical relationships between breeds, showed high haplotype sharing between the Welsh breeds and the Finnsheep and Scottish Blackface breeds (Additional file 6: Figure S6A). As expected, the Welsh breeds had extensive haplotype sharing with other UK breeds and breeds from Australia, New Zealand and North America. Out of the 18 Welsh breeds, only six had extensive haplotype sharing with Irish breeds (Additional file 7: Table S1). Furthermore, ten Welsh breeds had high degrees of haplotype sharing with breeds from Spain, Italy or Portugal (Additional file 7: Table S1), while the Black Welsh Mountain had extensive haplotype sharing (*r* = 0.374) with the Old Norwegian Spaelsau breed. Interestingly, six Welsh breeds (Kerry Hill, Badger Faced, Hill Flock Welsh Mountain, Dolgellau Welsh Mountain, Tregaron and Talybont Welsh Mountain) did not have a high degree of haplotype sharing with any of the non-Welsh UK breeds (Additional file 7: Table S1). At the 25–50Kb resolution, which presumably reflects more recent historical relationships, the International Sheep Genome Consortium breeds with highest haplotype sharing with Welsh breeds were the French Lacaune (Milk and Meat), US Gulf Coast Native and several Australian breeds of European origin (Additional file 6: Figure S6B).

### Population genetic structure of the 18 Welsh breeds

**Fig. 4:**
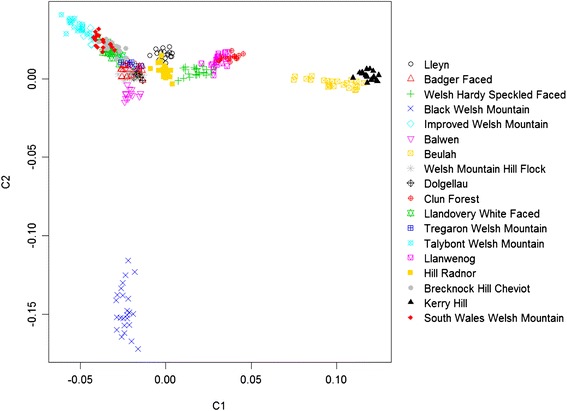
Clustering of individuals from 18 native Welsh sheep breeds based on multi-dimensional scaling analysis of genotype data. The image shows a plot of the first two components, revealing clustering of a central group of mountain breeds

To identify population structure within the native Welsh breeds we performed both model based (i.e. STRUCTURE, Additional file 8: Figure S7) and assumption free MDS clustering analyses on a data set consisting solely of the 18 Welsh breeds (Fig. 4). Model-based STRUCTURE analysis was performed with assumed numbers of populations (K) between one and 18. Several interesting patterns of clustering were observed between *K* = 2, which separated the Beulah and Kerry Hill breeds, and *K* = 18, which revealed breed specific clustering for all breeds. First, both types of analyses identified a group of mountain breeds, which was differentiated from the Beulah, Kerry Hill and Black Welsh Mountain breeds. This was visible in the STRUCTURE output as separation of these groups at the *K* = 3 level (Additional file 8: Figure S7) and on the MDS plot (Fig. 4) as a central cluster (the mountain breeds), with two smaller distinct clusters (Black Welsh and Beulah/Kerry Hill). This pattern was also consistent with pairwise *F*_ST_ values_,_ with the highest value observed between the Black Welsh Mountain and Kerry Hill breeds (*F*_ST_ = 0.201) and the lowest between the Llandovery White Faced and Improved Welsh Mountain breeds (*F*_ST_ = 0.020) (Additional file 9: Table S2). By comparison, pairwise *F*_ST_ ranged from 0.099 to 0.186 between the Welsh breeds and another common British breed (Scottish Texel; 359,000 UK Texel ewes as of 2012), and from 0.160 to 0.257 between the Welsh breeds and the Asian breed used as an outgroup in phylogenetic analysis (Indian Garole) [43]. Second, STRUCTURE analysis with *K* = 4 resulted in the detection of a cluster formed by the Clun Forest and Llanwenog breeds, which were differentiated from the remaining mountain breeds (Additional file 8: Figure S7). This pattern was also detected through a neighbour joining phylogenetic analysis based on the Welsh breed data only (Additional file 10: Figure S8).

Several of the mountain breeds within the defined central cluster had pairwise *F*_ST_ values that were lower than those between different geographic sub-populations of the same breed from the International Sheep Genome Consortium data set. For example, the pairwise *F*_ST_ between Brown and White East-Friesian International Sheep Genome Consortium individuals was 0.080, whereas between the Tregaron and Dolgellau Welsh Mountain breeds this value was 0.025. Similarly, pairwise *F*_ST_ of 0.042 was observed between New Zealand and German Texel individuals, but only 0.020 between the Improved Welsh Mountain and Llandovery White Faced breeds, which was also supported by the high extent of haplotype sharing (*r* > 0.45 at 0–10Kb) (Additional file 6: Figure S6A) between the latter two breeds. Of interest was the Black Welsh Mountain breed, which had consistently high pairwise *F*_ST_ with all Welsh breeds (from 0.102 with the Tregaron Welsh Mountain to 0.201 with Kerry Hill), as well as a relatively low effective population size (*N*_*e*_ = 89).

### Historical demographic trends

**Fig. 5:**
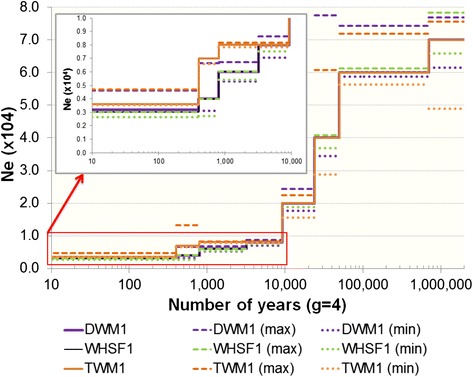
Demographic inference for three individual sheep of different native Welsh breeds. The breeds used were the Dolgellau Welsh Mountain (DWM1), Tregaron Welsh Mountain (TWM1) and Welsh Hardy Speckled Faced (WHSF1). In each demographic plot, a standard mutation rate of 1×10^−8^ has been scaled by the appropriate false negative error rate for each animal (Additional file 11: Table S3). Demographic trends were inferred using the HHn method (see Methods)

The demographic histories inferred by both the PSMC (Additional file 1: Figure S1) and HHn (Fig. 5; Additional file 11: Table S3) methods were all broadly similar using either method and for all three individuals sequenced. The PSMC method is not recommended for demographic inference in recent time therefore we relied on the HHn for the most recent 1000 years demography [33]. Both methods reflect a very large ancestral effective population size ~1 M years ago which then decreased rapidly until the presumed time of sheep domestication around 10,000 years ago. Interestingly, the PSMC method suggested a possible rebound in *N*_*e*_ around 100,000 years ago when the common ancestors of the sequenced individuals were wild sheep (Additional file 1: Figure S1). As both the PSMC and HHn methods are affected by assumptions about the rates of mutation and sequencing error (see Methods), we tested the sensitivity of the demographic trends detected using these methods. As expected, doubling the mutation rate reduced the absolute value of *N*_*e*_, but resulted in a demographic trend similar to that detected with the lower mutation rate (PSMC results in Additional file 2: Figure S2).

## Discussion

The advent of high throughput genotyping arrays has greatly facilitated the study of genetic diversity. In livestock species, such studies provide a powerful resource to determine the genetic basis of economically important traits segregating within or between breeds [44–46]. Whole-genome genotyping has also informed our understanding of livestock population genetic structure, origins and migration, as well as the movement of human populations linked to these processes [47–49]. Whilst much effort has been devoted to uncovering genetic differences, relationships and population structure within dominant commercial breeds, the less widely used local breeds are generally understudied. In sheep, one exception to this pattern is the Soay breed, an isolated island breed, which has been extensively studied as a model for how recent admixture, isolation and selection affect a population [50, 51]. Local breeds could, however, be important gene pools for adaptive traits and can be used to ensure the efficacy of future geographically-targeted breeding programmes. To this end, we outlined the population structure of 18 breeds native to Wales in the context of 74 breeds surveyed by the International Sheep Genome Consortium [52]. The program STRUCTURE and MDS were used to identify subpopulations and levels of admixture. We also performed phylogenetic analysis and estimated rates of LD decay and haplotype sharing for the native Welsh and worldwide breeds. In addition, we determined patterns of demography from the whole genome sequence of three individuals of different mountain breeds and estimated recent effective population sizes from LD.

### Ancestry of the native Welsh breeds

Analysis based on a genome wide set of loci was clearly able to distinguish Welsh breeds from those developed in other parts of Europe and beyond. Individuals from Welsh breeds grouped together (e.g. Fig. 2) and were often more closely related to each other than to other European breeds. None the less, the results of MDS and STRUCTURE analyses indicate that the native Welsh breeds show close genetic similarity to other European breeds (Figs. 1 and 3). Within the European population, the predominant clusters were the Texel and Merino groups. These breeds are commercially valuable, and their close association with British and Welsh breeds is not surprising [53, 54]. In addition, the Texel breed was improved in the early 20th century through crossing with breeds of British origin, further corroborating its relationship with UK native breeds [55].

The results of haplotype sharing analysis suggested common ancestry within the European population. The Texel and Merino breeds once again appeared to have high levels of co-ancestry with other European breeds, including the native Welsh breeds. A logical next step in further understanding the true relationship between the Welsh, Texel and Merino breeds, which is not completely resolved based on our phylogenetic and haplotype analyses, would be performing higher resolution analyses using whole-genome sequences of individuals from these breeds. High levels of haplotype sharing were observed between the Welsh breeds and breeds from Australia and America at the highest resolution studied (Additional file 6: Figure S6A), likely reflecting historical exchange of livestock breeds between Wales, Europe, Australia and America.

Many of the Welsh breeds showed high levels of 'fine-scale' (i.e. 0–10Kb) haplotype sharing with breeds from France, Spain, Portugal and Italy. These results may reflect the historical migration of people and livestock from continental Europe to the UK, which has been documented as far back as Roman times. In addition, fine-scale haplotype sharing also suggested a distant historical relationship between Welsh breeds, including the Improved Welsh Mountain and Kerry Hill and the Scandinavian Finnsheep. Whilst these relationships are of interest, they are not necessarily connected to human migration to Wales from Scandinavia because records show that crosses with the Finnsheep breed have commonly been used to increase the fertility of other breeds [56]. However, a further historical link between Wales and Scandinavia was suggested by the extensive fine-scale haplotype sharing between the Black Welsh Mountain and the Old Norwegian Spaelsau breed. Co-ancestry between the Old Norwegian Spaelsau and several Welsh breeds was also supported by relatively low pairwise *F*_ST_ values, notably with the Improved Welsh, Tregaron Welsh Mountain and Badger Faced Welsh Mountain (*F*_ST_ = 0.065, 0.060 and 0.069, respectively). This is also consistent with presumed patterns of human migration [57]. Taken together, these findings strongly suggest historical ties between breeds from Wales and Scandinavia, possibly dating as far back as the Viking invasion [58, 59].

Differences in haplotype sharing were also observed among Welsh mountain breeds. Whilst the Improved and Hill Flock Welsh Mountain breeds had high levels of fine-scale haplotype sharing with breeds from Scandinavia, Spain, Portugal and Italy (Additional file 6: Figure S6A, Additional file 7: Table S1), the South Wales Welsh Mountain breed did not, suggesting that the latter breed has a somewhat distinct ancestry.

As expected, high levels of haplotype sharing were observed at all scales between the Welsh and other British or Irish breeds (Additional file 6: Figure S6). Perhaps of particular note is the high level of putative co-ancestry between the Irish Galway breed and the Welsh Lleyn (*r* > 0.43 in the 0–10Kb range), a pattern that was also consistent with results from our phylogenetic analysis (Fig. 2), as well as with the presumed common origin of these breeds documented in the 18th century [16, 60].

### Population genetic structure of the 18 Welsh breeds

Analysis of the Welsh breeds revealed substantial variation in pairwise *F*_ST_ values between breeds (range = 0.020–0.201 with mean = 0.107). This is higher than levels observed in other studies in sheep (mean *F*_ST_ = 0.061) [61] but lower than that found in studies of local (French) breeds of cattle (mean *F*_ST_ = 0.190) [62]. Both STRUCTURE and MDS analyses identified a central group of thirteen breeds as well as two separate clusters, one comprising the Kerry Hill and Beulah individuals and the other the Black Welsh Mountain breed (Figs. 3 and 4, Additional file 8: Figure S7).

In addition, the phylogenetic tree topology indicated potential shared ancestry of the Welsh Hardy Speckled Faced breed (Fig. 2), the Kerry Hill and Beulah breeds. This is consistent with the hypothesis that the Welsh Hardy Speckled Faced was derived by crossing the Kerry Hill with an unknown Welsh Mountain breed [55].

The central cluster included the majority of Welsh mountain breeds. Pairwise *F*_ST_ values among these breeds tended to be relatively low (*F*_ST_ <0.107). This is consistent with a common origin for the majority of the mountain breeds and raises questions about the levels of differentiation required to classify different groups as separate breeds rather than “types”.

The strong differentiation of the Black Welsh Mountain breed (Figs. 3 and 4) was not surprising. Welsh mountain individuals with black colouring are known to have occurred as far back as medieval times, when these individuals were in high demand because of their coloured fleece [63]. Whilst the other mountain breeds have presumably arisen through selection for survival in the harsh mountain environment, the Black Welsh was formed on the basis of fleece colour and aesthetics [63]. This may have contributed to their reduced effective population size and higher rate of inbreeding relative to most other Welsh breeds (Table 1).

### Single nucleotide polymorphism, diversity and linkage disequilibrium within Welsh breeds

The Black Welsh Mountain, Kerry Hill, and Balwen breeds had the lowest recent *N*_*e*_. This could have resulted from population bottlenecks, and there is some evidence to support this for the Balwen breed. Records show that the breed was brought near extinction by the exceptionally harsh winter of 1946/1947, which is believed to have left only one breeding ram remaining [64, 65]. Consequently, the rare breeds survival trust (RBST) categorises the Balwen as “at risk”, with a population of between 900 and 1500 breeding females [66]. It is possible that the Black Welsh Mountain and Kerry Hill breeds have suffered similar reductions in numbers, and this may also account for the high levels of inbreeding estimated for these breeds. In addition to the Balwen, the Hill Radnor breed is also classified as being “at risk” [10]. Our estimate of *N*_*e*_ for this breed was 198, which is below the mean of 316 (Table 1). Several of the breeds in our sample set are now recovering after periods of featuring on the RBSTs watch list of rare and endangered breeds [10]. These include the Black Welsh Mountain, Kerry Hill, Lleyn, Llanwenog and South Wales Welsh Mountain. All of these breeds had *N*_*e*_ lower than the average. This, in addition to the population stratification we detected, will have implications for future genome-wide association studies [67].

### Historical demographic trends

Demographic inference using the HHn and PSMC approaches indicated a steep reduction in the effective size of wild sheep populations prior to domestication (Fig. 5, Additional file 1: Figure S1). This reduction coincides with the Last Glacial Maximum (20,000 to 30,000 years ago [68]) and may possibly be a result of reduced habitat range or geographic isolation of some wild sheep populations. The continued reductions in *N*_*e*_ from around 12,000 years ago would be expected as a result of domestication followed by breed development, as observed in cattle [34]. Although the inferred demographies are reassuringly similar, the PSMC approach indicated that after an initial reduction in the ancestral wild population, there was a slight rebound in *N*_*e*_ prior to further steep decrease. It is possible that this is not a true increase in the *N*_*e*_ but rather a signal of some sub-populations beginning to diverge, but with continued migration between them [33]. The HHn inference shows a large confidence interval across this period for the Tregaron Welsh Mountain and Dolgellau Welsh Mountain breeds and therefore is not in disagreement with the PSMC inference of a possible rebound in *N*_*e*_, although this is not the case for the Welsh Hardy Speckled Faced individual. We are currently collecting whole-genome sequence data from a larger number of Welsh breeds to further clarify these findings.

### Conclusions

Our study used genotype data from 18 Welsh breeds to provide a first glimpse into the population structure of native sheep. We identified four subpopulations, with many of the Welsh mountain breeds forming a relatively homogeneous group. Pairwise *F*_ST_ values for some of these breeds were lower than previously reported values between members of the same breed from different geographical locations. These findings have implications for the design of future genome-wide association studies, as failing to correctly account for population structure may lead to false positive results [69]. For example, our data suggest that it may be possible to group animals from the different mountain breeds within the same association mapping population. This is significant as it could potentially increase the statistical power to detect polymorphisms with minor effects [70].

We identified breeds with low effective population sizes and high levels of inbreeding, potentially informing monitoring and restoration of genetic diversity through planned breeding strategies. Our results complement research collected by organisations such as the RBST, which plays a key role in conserving these smaller breeds.

Finally, analysis of our data combined with those from the International Sheep Genome Consortium revealed a common ancestry of Welsh and other European breeds. The commercial Texel and Merino breeds appear to be key contributors to the European population, presumably because these breeds have been included in most breeding programmes. Further understanding of the relationships between breeds within Wales, the UK and the rest of Europe will facilitate the progress of genomics assisted breeding strategies, with the overall aims of lower cost, increased efficiency, improved livestock health and monitoring of inbreeding.

## Ethics statement

All blood samples from the Welsh breeds were collected according to UK Home Office guidelines and in line with the Animals (Scientific Procedures) Act 1986.

## Availability of supporting data

The Welsh 50K SNP data set is available from the Dryad repository and can be accessed at http://dx.doi.org/10.5061/dryad.j3k0q.

## Additional files

Additional file 1: Figure S1. Historical demographic trends for three Welsh sheep breeds using a pairwise sequentially Markovian coalescent (PSMC) model. One individual was used for each breed: Dolgellau Welsh Mountain (A), Tregaron Welsh Mountain (B) and Welsh Hardy Speckled Faced (C). For each breed, a standard mutation rate of 1 × 10^−8^ has been scaled by the estimated false negative error rate for each sequenced animal (see Additional file 11: Table S3). Confidence intervals are shown using PSMC bootstrapping. Additional file 2: Figure S2. Historical demographic trends for the Welsh Hardy Speckled Faced sheep breed. Figure based on inference with the pairwise sequentially Markovian coalescent (PSMC) model with an assumed mutation rate of 2 × 10^−8^ (all other parameters were the same as for Additional file 1: Figure S1). Additional file 3: Figure S3. Decay of linkage disequilibrium (LD) within and across 18 Welsh sheep breeds. LD was quantified as pairwise genotypic correlation (*r* ^*2*^) among common (MAF ≥ 0.10) single-nucleotide polymorphisms. The Soay and Australian Merino breeds were included for comparison based on their relatively slow and rapid LD decay, respectively, as detected in a previous study [11]. Additional file 4: Figure S4. Population structure of the combined data set determined by model based clustering. STRUCTURE runs were performed on a combined data set of the Welsh breeds and the International Sheep Genome Consortium HapMap data. The analysis was run with assumed numbers of populations (K) between 1 and 7. The figure shows clustering results at K = 4. Additional file 5: Figure S5. Neighbour-joining phylogenetic tree of sheep breeds from America, Europe, Australia and Wales (red). Additional file 6: Figure S6. Haplotype sharing between Welsh sheep breeds and their most related worldwide breeds. Haplotype sharing was calculated for markers in four distance intervals A) 0–10Kb, B) 10–25Kb, C) 25–50Kb and D) 50–100Kb. The ten International Sheep Genome Consortium HapMap breeds having the highest haplotype sharing with any Welsh breed are indicated with asterisks. Additional file 7: Table S1. Haplotype sharing between the 18 Welsh and 10 top worldwide breeds summarised by geographical origin. Additional file 8: Figure S7. Population structure of 18 Welsh sheep breeds determined by model based clustering. The analysis was run with assumed numbers of populations (K) between 1 and 18. The figure shows the clustering results for K = 2–5. Additional file 9: Table S2. Pairwise genetic differentiation (*F*_ST_) between Welsh, British and Asian breeds, Scottish Texel and Indian Garole. Additional file 10: Figure S8. Neighbour-joining phylogenetic tree of 18 Welsh sheep breeds and an Asian outgroup. 1) Kerry Hill, 2) Welsh Hardy Speckled Faced, 3) Hill Radnor, 4) Black Welsh Mountain, 5) Indian Garole (outgroup), 6) Balwen, 7) Badger Faced, 8) South Wales Welsh Mountain, 9) Brecknock Hill Cheviot, 10) Llandovery White Faced, 11) Improved Welsh Mountain, 12) Talybont Welsh Mountain, 13) Hill Flock Welsh Mountain, 14) Dolgellau Welsh Mountain, 15) Tregaron Welsh Mountain, 16) Lleyn, 17) Llanwenog, 18) Clun Forest, 19) Beulah. Additional file 11: Table S3. Summary statistics for the whole genome sequence of three Welsh sheep after strict quality control.

## Abbreviations

BWA: Burrows-wheeler aligner
GWAS: Genome wide association studies
HHn: Haplotype homozygosity method
LD: Linkage disequilibrium
MAF: Minor allele frequency
MCMC: Markov chain Monte Carlo
MDS: Multidimensional scaling analysis
PSMC: Pairwise sequentially Markovian coalescent model
QTL: Quantitative trait locus
RBST: Rare breed survival trust
RoH: Runs of homozygosity
SNP: Single nucleotide polymorphism
