# Supplementary figures and images for: Population structure and history of the Welsh sheep breeds determined by whole genome genotyping
Source: BMC Genet. 2015 Jun 20;16:65. doi: 10.1186/s12863-015-0216-x (PMC4474581; doi:10.1186/s12863-015-0216-x)

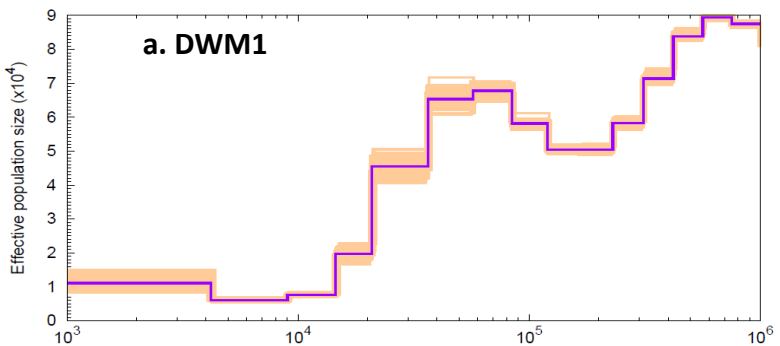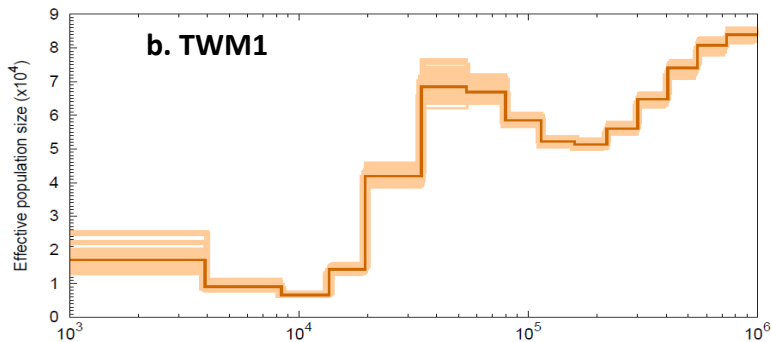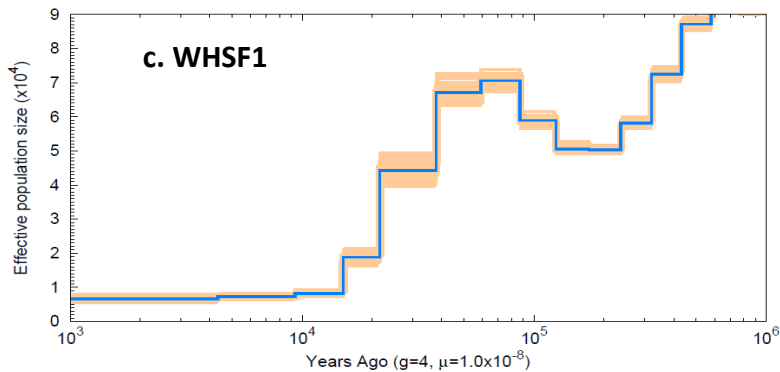

Supplement: Additional file 1: Figure S1. — Historical demographic trends for three Welsh sheep breeds using a pairwise sequentially Markovian coalescent (PSMC) model. One individual was used for each breed: Dolgellau Welsh Mountain (A), Tregaron Welsh Mountain (B) and Welsh Hardy Speckled Faced (C). For each breed, a standard mutation rate of 1 × 10^−8^ has been scaled by the estimated false negative error rate for each sequenced animal (see Additional file 11: Table S3). Confidence intervals are shown using PSMC bootstrapping. [file 12863_2015_216_MOESM1_ESM.pdf]

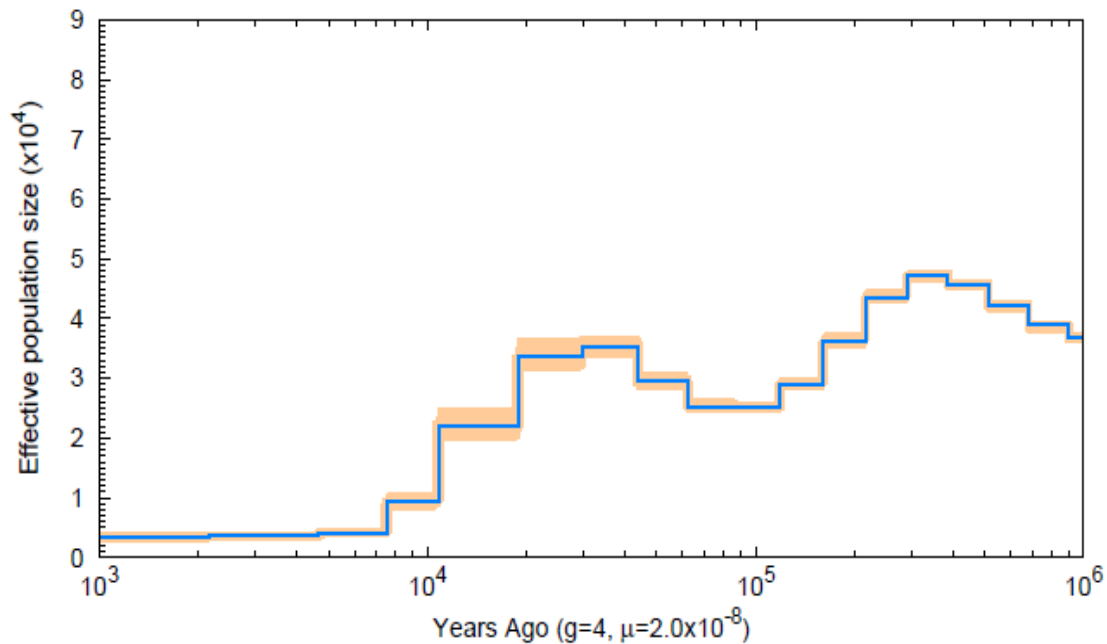

Supplement: Additional file 2: Figure S2. — Historical demographic trends for the Welsh Hardy Speckled Faced sheep breed. Figure based on inference with the pairwise sequentially Markovian coalescent (PSMC) model with an assumed mutation rate of 2 × 10^−8^ (all other parameters were the same as for Additional file 1: Figure S1). [file 12863_2015_216_MOESM2_ESM.pdf]

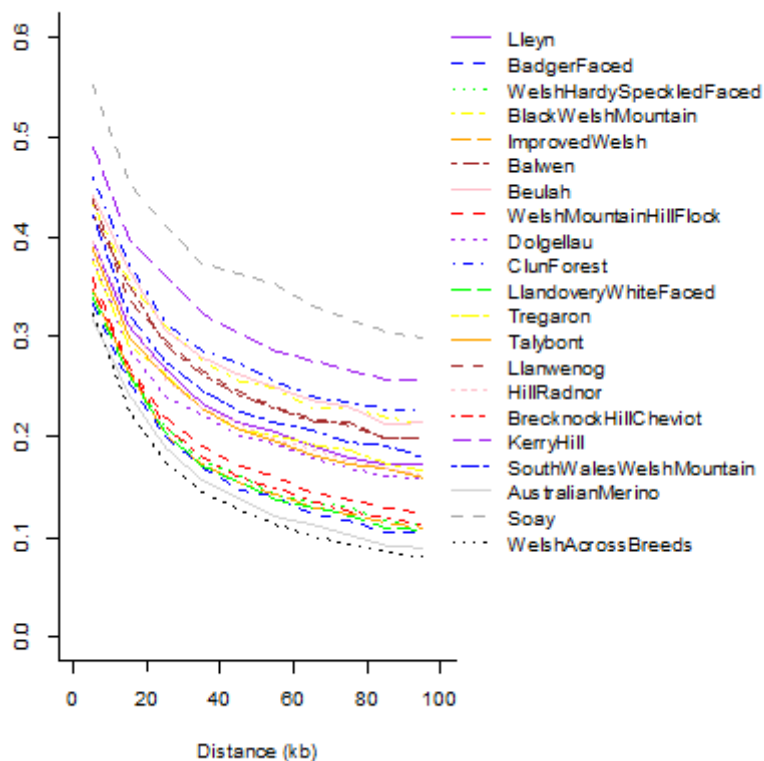

Supplement: Additional file 3: Figure S3. — Decay of linkage disequilibrium (LD) within and across 18 Welsh sheep breeds. LD was quantified as pairwise genotypic correlation (r 2) among common (MAF ≥ 0.10) single-nucleotide polymorphisms. The Soay and Australian Merino breeds were included for comparison based on their relatively slow and rapid LD decay, respectively, as detected in a previous study [11]. [file 12863_2015_216_MOESM3_ESM.pdf]

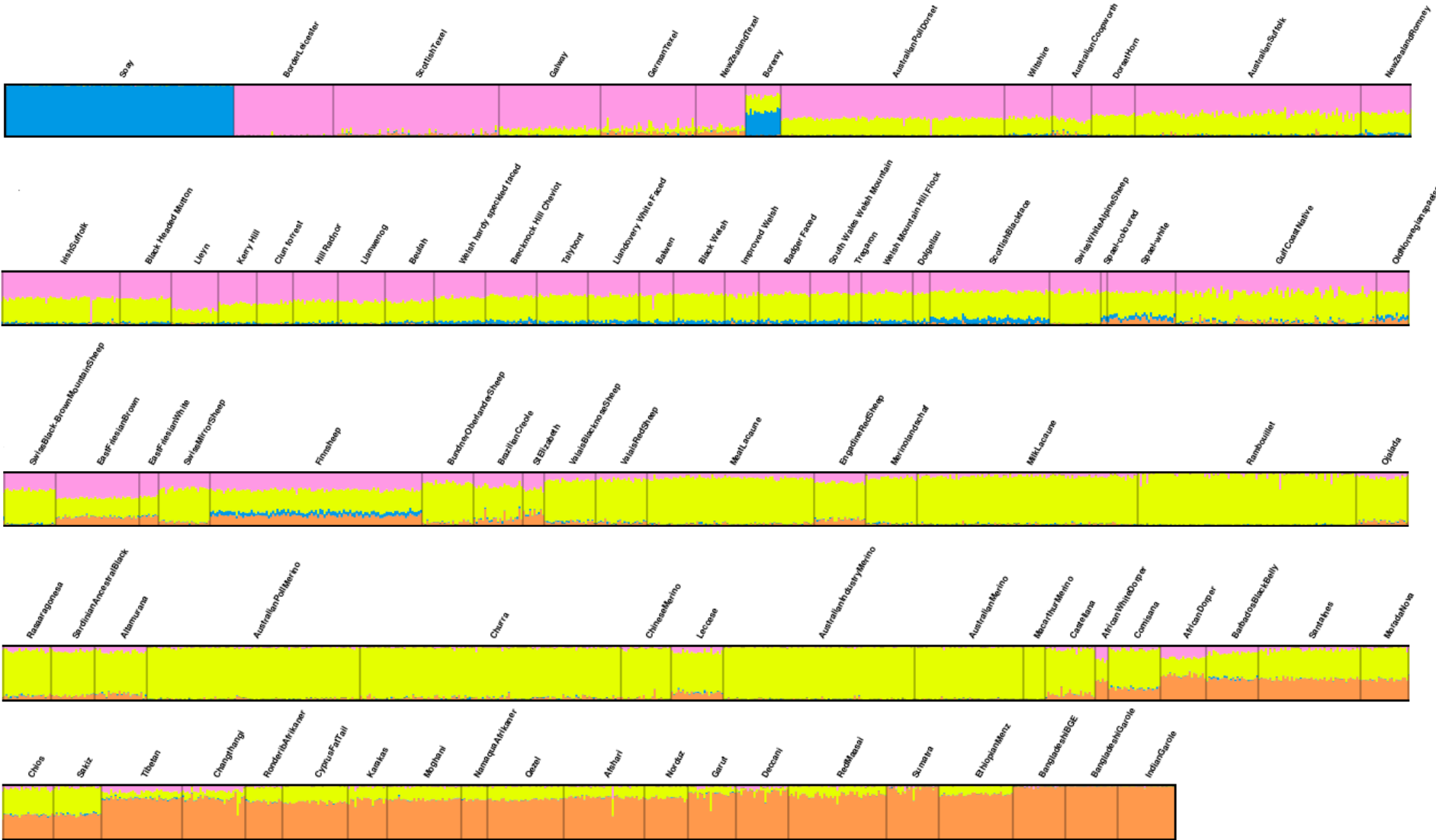

Supplement: Additional file 4: Figure S4. — Population structure of the combined data set determined by model based clustering. STRUCTURE runs were performed on a combined data set of the Welsh breeds and the International Sheep Genome Consortium HapMap data. The analysis was run with assumed numbers of populations (K) between 1 and 7. The figure shows clustering results at K = 4. [file 12863_2015_216_MOESM4_ESM.pdf]

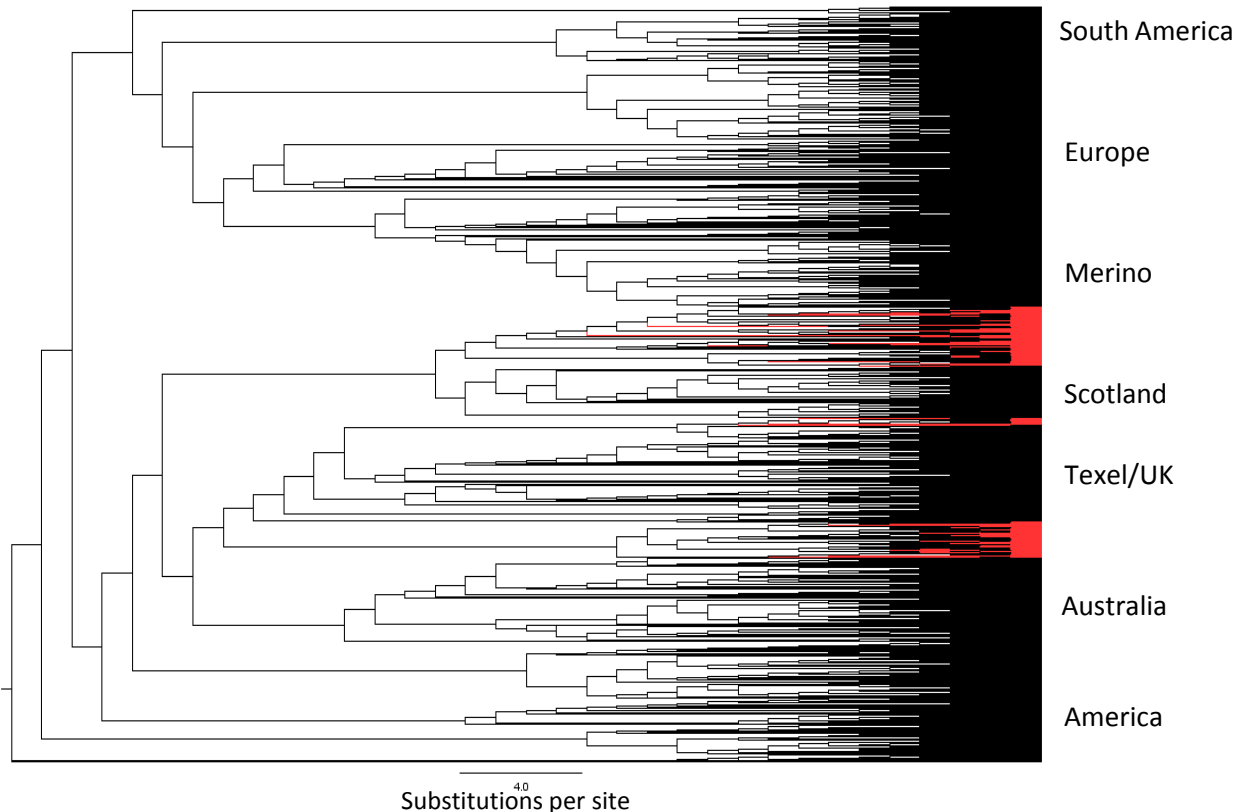

Supplement: Additional file 5: Figure S5. — Neighbour-joining phylogenetic tree of sheep breeds from America, Europe, Australia and Wales (red). [file 12863_2015_216_MOESM5_ESM.pdf]

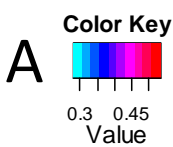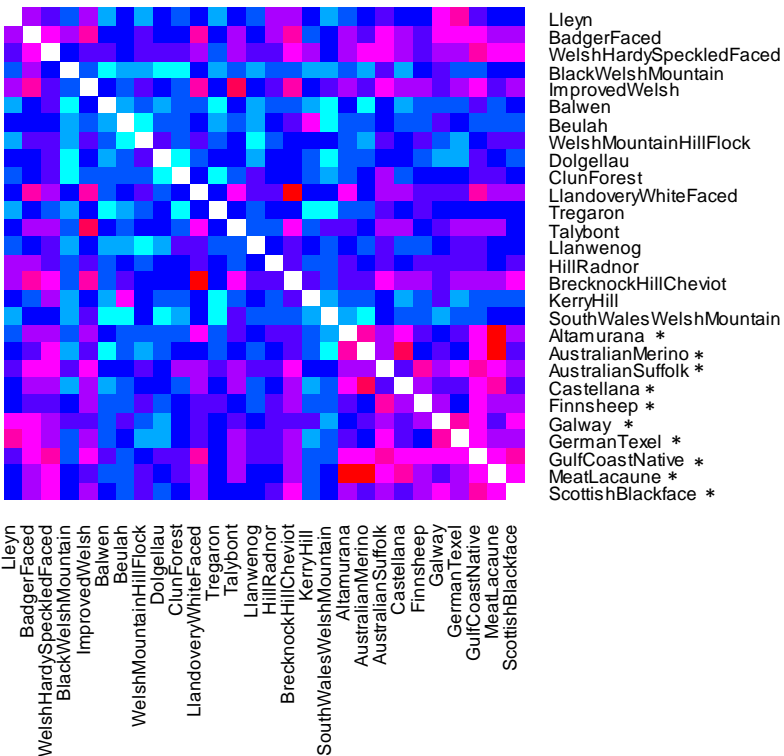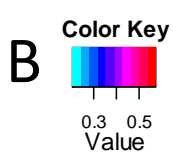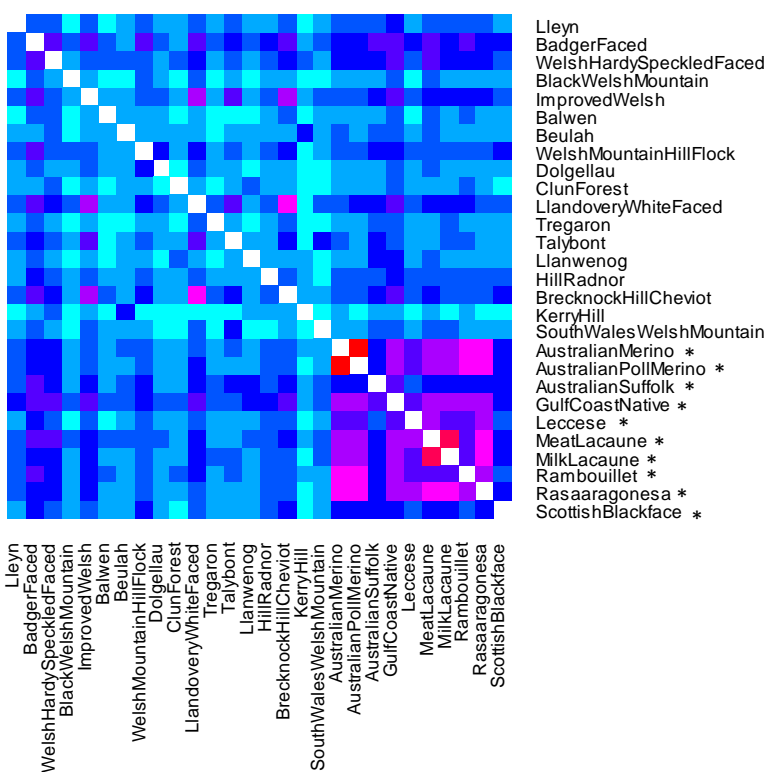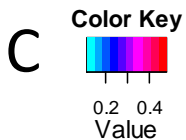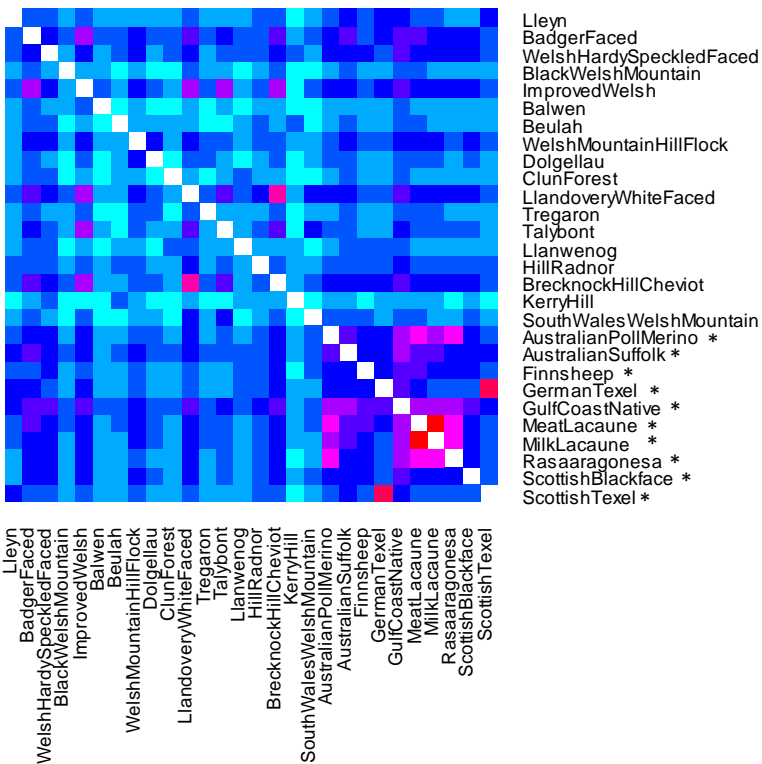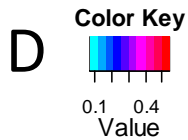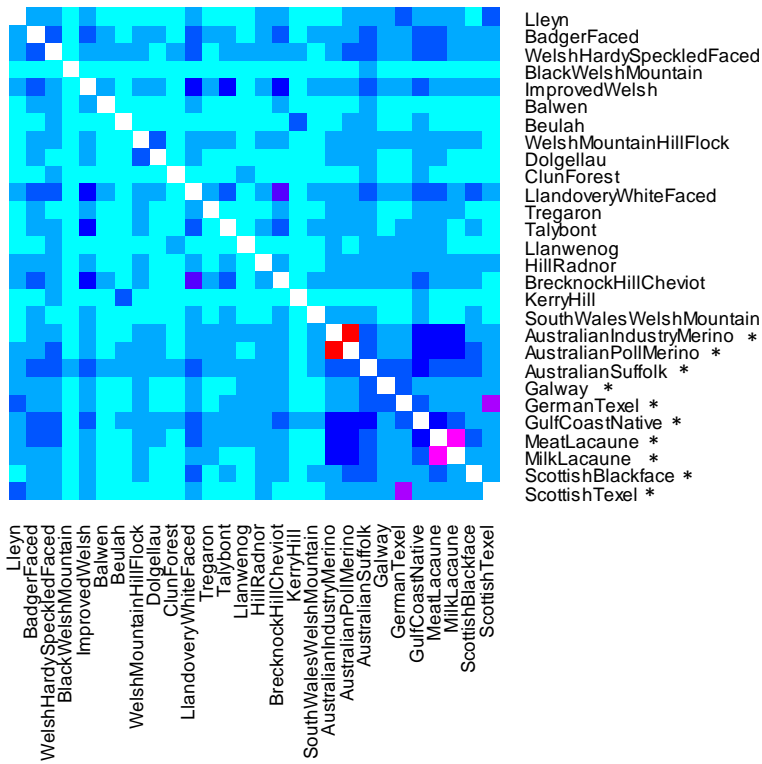

Supplement: Additional file 6: Figure S6. — Haplotype sharing between Welsh sheep breeds and their most related worldwide breeds. Haplotype sharing was calculated for markers in four distance intervals A) 0–10Kb, B) 10–25Kb, C) 25–50Kb and D) 50–100Kb. The ten International Sheep Genome Consortium HapMap breeds having the highest haplotype sharing with any Welsh breed are indicated with asterisks. [file 12863_2015_216_MOESM6_ESM.pdf]

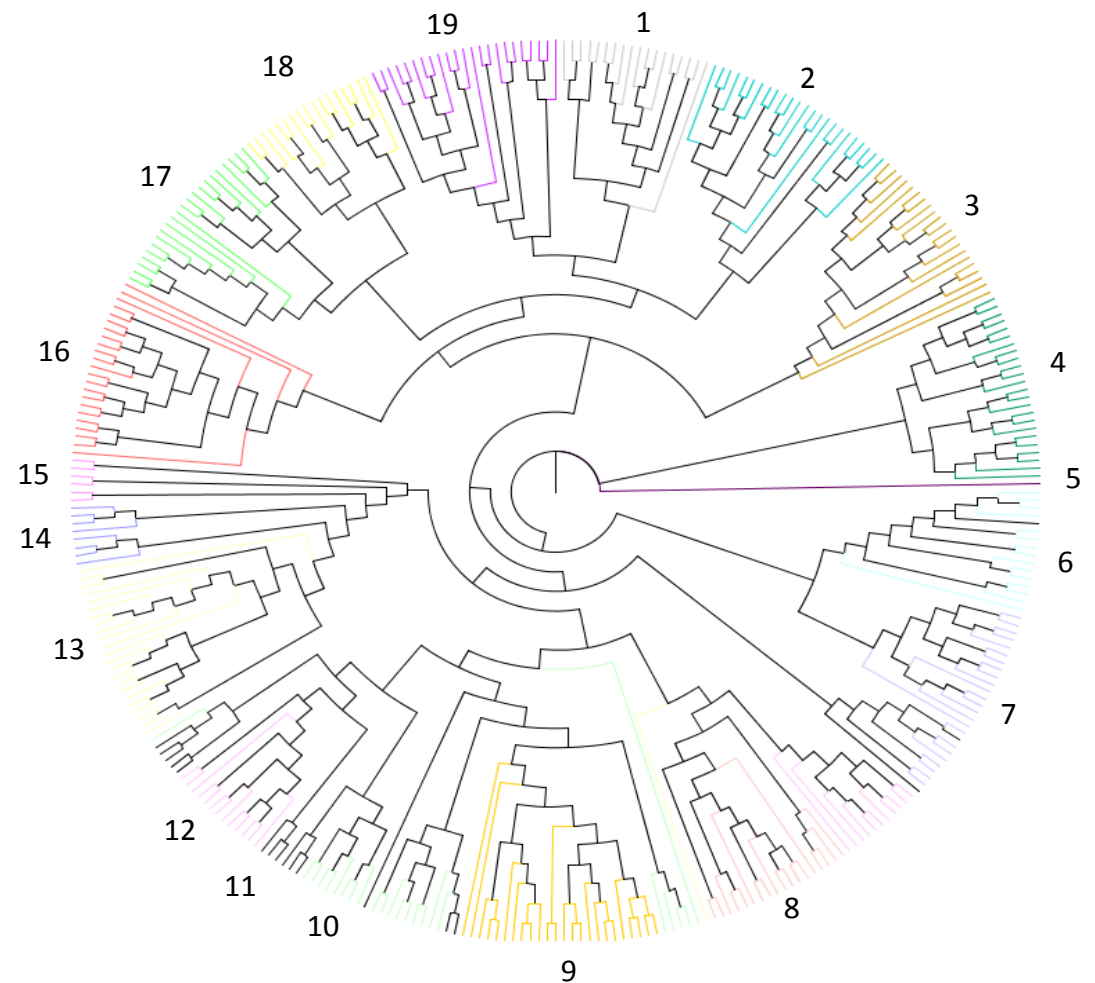

Supplement: Additional file 10: Figure S8. — Neighbour-joining phylogenetic tree of 18 Welsh sheep breeds and an Asian outgroup. 1) Kerry Hill, 2) Welsh Hardy Speckled Faced, 3) Hill Radnor, 4) Black Welsh Mountain, 5) Indian Garole (outgroup), 6) Balwen, 7) Badger Faced, 8) South Wales Welsh Mountain, 9) Brecknock Hill Cheviot, 10) Llandovery White Faced, 11) Improved Welsh Mountain, 12) Talybont Welsh Mountain, 13) Hill Flock Welsh Mountain, 14) Dolgellau Welsh Mountain, 15) Tregaron Welsh Mountain, 16) Lleyn, 17) Llanwenog, 18) Clun Forest, 19) Beulah. [file 12863_2015_216_MOESM10_ESM.pdf]
