# Supplementary material for: Population structure and history of the Welsh sheep breeds determined by whole genome genotyping
Source: BMC Genet. 2015 Jun 20;16:65. doi: 10.1186/s12863-015-0216-x (PMC4474581; doi:10.1186/s12863-015-0216-x)
Supplement: Additional file 7: Table S1. — Haplotype sharing between the 18 Welsh and 10 top worldwide breeds summarised by geographical origin. [file 12863_2015_216_MOESM7_ESM.pdf]

Supplementary Table 1. **Haplotype sharing between the 18 Welsh and 10 top worldwide breeds summarised by geographical origin.**

	European breeds							USA	Iranian	New Zealand/ Australian
	Scandinavian	Spanish/Portuguese/Italian	Irish	French	UK (non-Welsh)	German	Swiss			
Beulah			+		+		+			+
Kerry Hill		+	+							+
Black Welsh Mountain	+		+	+	+					
Clun Forest				+	+			+		+
Llanwenog		+			+	+		+		+
Badger Faced			+				+	+		+
Hardy Speckled Faced		+		+	+	+		+		+
Improved Welsh Mountain	+	+			+	+		+		+
Balwen		+			+			+		+
Hill Flock Welsh Mountain	+	+		+						+
Dolgellau Welsh Mountain		+		+				+		+
Llandovery White Faced		+			+		+	+		+
Tregaron Welsh Mountain	+			+				+	+	
Talybont Welsh Mountain		+		+		+		+		+
Hill Radnor		+	+		+			+		+
Brecknock Hill Cheviot	+				+	+		+		+
South Wales Welsh Mountain				+	+					+
Lleyn			+		+	+		+		+

The International Sheep Genome Consortium (ISCG) breeds with the highest levels of haplotype sharing (top 10) with each of the Welsh breeds are summarised by geographical origin, based on 0-10Kb resolution.
