# Supplementary material for: Population structure and history of the Welsh sheep breeds determined by whole genome genotyping
Source: BMC Genet. 2015 Jun 20;16:65. doi: 10.1186/s12863-015-0216-x (PMC4474581; doi:10.1186/s12863-015-0216-x)
Supplement: Additional file 8: Figure S7. — Population structure of 18 Welsh sheep breeds determined by model based clustering. The analysis was run with assumed numbers of populations (K) between 1 and 18. The figure shows the clustering results for K = 2–5. [file 12863_2015_216_MOESM8_ESM.pdf]

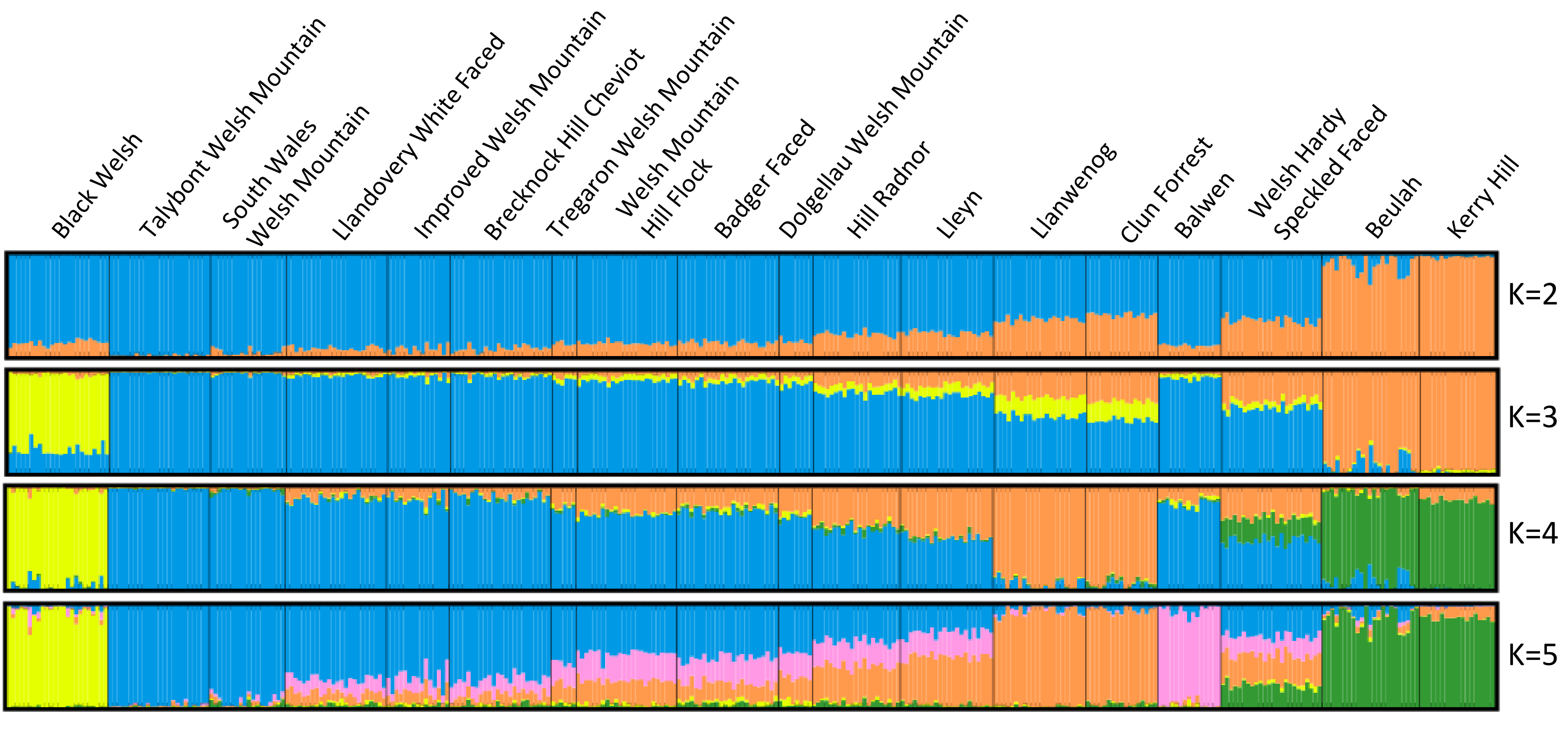
Black Welsh
Talybont Welsh Mountain
South Wales Welsh Mountain
Llandovery White Faced
Improved Welsh Mountain
Brecknock Hill Cheviot
Tregaron Welsh Mountain
Welsh Mountain Hill Flock
Badger Faced
Dolgellau Welsh Mountain
Hill Radnor
Lleyn
Llanwenog
Clun Forrest
Balwen
Welsh Hardy Speckled Faced
Beulah
Kerry Hill
K=2
K=3
K=4
K=5
