# Supplementary material for: Population structure and history of the Welsh sheep breeds determined by whole genome genotyping
Source: BMC Genet. 2015 Jun 20;16:65. doi: 10.1186/s12863-015-0216-x (PMC4474581; doi:10.1186/s12863-015-0216-x)
Supplement: Additional file 9: Table S2. — Pairwise genetic differentiation (F ST) between Welsh, British and Asian breeds, Scottish Texel and Indian Garole. [file 12863_2015_216_MOESM9_ESM.pdf]

Supplementary Table 2. Pairwise genetic differentiation (F_{ST}) between Welsh, British and Asian breeds, Scottish Texel and Indian Garole.

		1	2	3	4	5	6	7	8	9	10	11	12	13	14	15	16	17	18	19	20
**1**	Lleyn	0	0.078	0.082	0.144	0.072	0.130	0.141	0.097	0.088	0.130	0.080	0.072	0.109	0.123	0.108	0.085	0.159	0.126	0.202	0.099
**2**	Badger Faced	0.078	0	0.051	0.106	0.030	0.091	0.112	0.057	0.047	0.110	0.040	0.031	0.068	0.099	0.078	0.045	0.137	0.084	0.170	0.105
**3**	Welsh Hardy Speckled	0.082	0.051	0	0.118	0.047	0.108	0.102	0.068	0.057	0.106	0.056	0.043	0.084	0.097	0.084	0.060	0.113	0.098	0.177	0.108
**4**	Black Welsh Mountain	0.144	0.106	0.118	0	0.104	0.162	0.177	0.126	0.117	0.174	0.113	0.102	0.140	0.163	0.146	0.118	0.201	0.155	0.234	0.171
**5**	Improved Welsh Mountain	0.072	0.030	0.047	0.104	0	0.085	0.109	0.054	0.044	0.104	0.020	0.022	0.036	0.095	0.074	0.026	0.132	0.074	0.168	0.100
**6**	Balwen	0.130	0.091	0.108	0.162	0.085	0	0.170	0.116	0.105	0.168	0.104	0.090	0.133	0.155	0.137	0.108	0.193	0.146	0.223	0.161
**7**	Beulah	0.141	0.112	0.102	0.177	0.109	0.170	0	0.131	0.119	0.163	0.117	0.105	0.146	0.156	0.144	0.121	0.130	0.159	0.237	0.168
**8**	Hill Flock Welsh Mountain	0.097	0.057	0.068	0.126	0.054	0.116	0.131	0	0.039	0.127	0.065	0.040	0.091	0.116	0.096	0.070	0.155	0.103	0.186	0.125
**9**	Dolgellau Welsh Mountain	0.088	0.047	0.057	0.117	0.044	0.105	0.119	0.039	0	0.118	0.054	0.025	0.081	0.105	0.086	0.061	0.144	0.094	0.176	0.114
**10**	Clun Forest	0.130	0.110	0.106	0.174	0.104	0.168	0.163	0.127	0.118	0	0.113	0.102	0.141	0.129	0.135	0.116	0.176	0.155	0.232	0.157
**11**	Llandovery White Faced	0.080	0.040	0.056	0.113	0.020	0.104	0.117	0.065	0.054	0.113	0	0.027	0.055	0.102	0.082	0.023	0.139	0.082	0.174	0.108
**12**	Tregaron Welsh Mountain	0.072	0.031	0.043	0.102	0.022	0.090	0.105	0.040	0.025	0.102	0.027	0	0.057	0.092	0.071	0.034	0.129	0.074	0.160	0.099
**13**	Talybont Welsh Mountain	0.109	0.068	0.084	0.140	0.036	0.133	0.146	0.091	0.081	0.141	0.055	0.057	0	0.131	0.111	0.061	0.170	0.086	0.203	0.137
**14**	Llanwenog	0.123	0.099	0.097	0.163	0.095	0.155	0.156	0.116	0.105	0.129	0.102	0.092	0.131	0	0.126	0.106	0.171	0.145	0.221	0.149
**15**	Hill Radnor	0.108	0.078	0.084	0.146	0.074	0.137	0.144	0.096	0.086	0.135	0.082	0.071	0.111	0.126	0	0.087	0.163	0.127	0.205	0.135
**16**	Brecknock Hill Cheviot	0.085	0.045	0.060	0.118	0.026	0.108	0.121	0.070	0.061	0.116	0.023	0.034	0.061	0.106	0.087	0	0.144	0.094	0.180	0.113
**17**	Kerry Hill	0.159	0.137	0.113	0.201	0.132	0.193	0.130	0.155	0.144	0.176	0.139	0.129	0.170	0.171	0.163	0.144	0	0.184	0.257	0.186
**18**	South Wales Welsh Mountain	0.126	0.084	0.098	0.155	0.074	0.146	0.159	0.103	0.094	0.155	0.082	0.074	0.086	0.145	0.127	0.094	0.184	0	0.215	0.154
**19**	Indian Garole	0.202	0.170	0.177	0.234	0.168	0.223	0.237	0.186	0.176	0.232	0.174	0.160	0.203	0.221	0.205	0.180	0.257	0.215	0	0.223
**20**	Scottish Texel	0.099	0.105	0.108	0.171	0.100	0.161	0.168	0.125	0.114	0.157	0.108	0.099	0.137	0.149	0.135	0.113	0.186	0.154	0.223	0

[a] Scottish Texel and Indian Garole breeds were included in this table to demonstrate F_{ST} ranges with non-Welsh European and Asian breeds.
