# Supplementary material for: Population structure and history of the Welsh sheep breeds determined by whole genome genotyping
Source: BMC Genet. 2015 Jun 20;16:65. doi: 10.1186/s12863-015-0216-x (PMC4474581; doi:10.1186/s12863-015-0216-x)
Supplement: Additional file 11: Table S3. — Summary statistics for the whole genome sequence of three Welsh sheep after strict quality control. [file 12863_2015_216_MOESM11_ESM.pdf]

Supplementary Table 3. Summary statistics for the whole genome sequence of three Welsh sheep after strict quality control.

Animal ID	Uniquely aligned (Mb)	Heterozygous false negative error rate[a]	Heterozygous false positive error rate	Mean read depth	Estimated true heterozygosity[b]
**DWM1[c]**	30,767	0.422	4.95×10^{-5}	12	0.002773
**TWM1[d]**	27,245	0.533	4.50×10^{-5}	11	0.002836
**WHSF1[e]**	32,368	0.364	6.24×10^{-5}	13	0.002816

[a] Calculated as the proportion of heterozygous sites that are missed in the sequence data.

[b] True heterozygosity estimated from the observed heterozygosity and corrected for false negative error rate: True heterozygosity = observed heterozygosity/[1-False Negative Error Rate]).

[c] Dolgellau Welsh Mountain, [d] Tregaron Welsh Mountain, [e] Welsh Hardy Speckled Faced
